# Ventilation With or Without Endotracheal Tube Leak in Prolonged Neonatal Asphyxia

**DOI:** 10.7759/cureus.17798

**Published:** 2021-09-07

**Authors:** Alexandros Douvanas, Maria Kalafati, Eleni Tamvaki, Alexandra Nieri, Apostolos Papalois, Christina Koulouglioti, Afrodite Aggelina, Elizabeth Papathanassoglou

**Affiliations:** 1 Pediatrics, Medical School, National and Kapodistrian University of Athens, Athens, GRC; 2 Faculty of Nursing, National and Kapodistrian University of Athens, Athens, GRC; 3 Children’s Intensive Treatment Unit (ITU), Great Ormond Street Hospital, London, GBR; 4 Translational Research and Training, Experimental, Educational and Research Centre, Elpen Pharmaceutical Co. Inc., Athens, GRC; 5 NHS Foundation Trust, University Hospitals Sussex, Brighton, GBR; 6 Emergency Medicine, School of Medicine, National and Kapodistrian University of Athens, Athens, GRC; 7 Internal Medicine: Critical Care, Faculty of Nursing, University of Alberta, Edmonton, CAN

**Keywords:** newborn piglet model, neonatal asphyxia, perinatal asphyxia, endotracheal tube leakage, neonatal resuscitation, birth respiratory support, swine models, return of spontaneous circulation

## Abstract

Background

Severe and prolonged asphyxia can result in either intrauterine fetal death and stillbirth or multiorgan failure in surviving neonates. Establishing effective ventilation is the primary aim of resuscitation in newborns with asphyxia. The objective of this study was to compare the outcome of resuscitation by applying an endotracheal tube (ETT) with less, an ETT with moderate, and an ETT with high leakage during mechanical ventilation in swine neonates after prolonged perinatal asphyxia.

Materials and methods

A prospective, randomized controlled laboratory study was performed. Thirty Landrace/large white pigs, aged one to four days and weighted 1.754±218 gr, were randomly allocated into three groups depending on the ETT size: Group C (less leak: ETT no 4.0, n=10); Group A (high leak: ETT no 3.0, n=10); and Group B (moderate leak: ETT no 3.5, n=10). Mechanical asphyxia was performed until their heart rate was less than 60 bpm or their mean arterial pressure was below 15 mmHg. All animals with return of spontaneous circulation (ROSC) were monitored for four hours for their hemodynamic parameters, arterial oxygen saturation, and lactate acid levels.

Results

We demonstrate that 70% of the surviving animals were ventilated with an ETT with a leak (no. 3.5 and 3). A statistically significant difference was noted in PO_2_ (p=0.032) between Group B (126.4±53.4 mmHg) compared to Group A (72.28±29.18 mmHg) and Group C (94.28±20.46 mmHg) as well as in the right atrial pressure (p<0.001) between Group C (4.5 mmHg) vs Groups A (2 mmHg) and B (2 mmHg) during ROSC time. Lactate levels were statistically significantly lower (p=0.035) in Group C (mean=0.92 ± 0.07mmol/L) as compared to Group A (mean=1.13 ± 0.1 mmol/L) and Group B (mean= 1.08 ± 0.07 mmol /L; p = 0.034) at 4h post-ROSC.

Conclusion

We provide preliminary evidence that ventilation with ETT with moderate leakage improves survival after 2h of ROSC, along with oxygenation and hemodynamic parameters, in a porcine model of neonatal asphyxia and resuscitation, compared to less leakage ETT.

## Introduction

In most cases, the transition to extrauterine life in term babies occurs naturally, without any assistance. However, due to the large number of births worldwide. the availability of adequate and timely intervention is life-saving for millions of newborns yearly. According to recent evidence, 5% of term infants require positive-pressure ventilation (PPV) to successfully transition, 2% require intubation, 0.1% receive cardiac compressions, and 0.05% compressions with adrenaline [1]. Perinatal asphyxia is defined as the interruption of blood flow or oxygen deprivation to and from the fetus around the time of birth. It may be caused by maternal or fetal hemorrhage, intermittent or acute umbilical cord compression, uterine rupture, or shoulder dystocia, which influence the supply of oxygenated blood to the fetus [2]. It is characterized by intermittent periods of hypoxia-ischemia that, if prolonged and intense enough, may cause irreversible damage to oxy-regulatory tissues such as hypoxic-ischemic encephalopathy (HIE). HIE may result in permanent neurological conditions such as seizure disorders, cerebral palsy, cognitive delays, and motor disabilities [3]. HIE is the leading cause of multiorgan failure in infants [4], and the second cause of neuro-disability worldwide [5]. Severe and prolonged asphyxia can result in either intrauterine fetal death and stillbirth or multiorgan failure in surviving neonates [6].

About 13-26 million newborns worldwide need some form of respiratory support at birth to move to extrauterine life, which remains the most critical step of neonatal resuscitation. Optimizing resuscitation is important to prevent morbidity and mortality from perinatal asphyxia [7]. Many newborn infants, even those who are asphyxiated, will respond to respiratory support alone. As a result, the primary focus of newborn resuscitation is establishing effective ventilation, whereas support of circulation is provided only for those who have persistent bradycardia or asystole. For the newborn infants who receive respiratory support at birth, it is suggested that an initial low oxygen concentration (21% to 30%) is preferable rather than high oxygen concentration (60% to 100%) [1]. Endotracheal intubation is recommended in the asphyxiated newborn for specific conditions using the appropriate size endotracheal tube (ETT) to ensure adequate ventilation with minimal leak and trauma to the airway [7]. Traditionally, uncuffed ETTs were used for endotracheal ventilation in neonates to prevent the development of subglottic stenosis due to mucosal injury by cuffed ETTs. There is significant evidence of airway damage in infants with tight ETTs during mechanical ventilation [8-9]; the ETT internal diameter, in millimeters, can be calculated as gestational age in weeks divided by 10. Typically, a 2.5 tube is appropriate for infants <1 kg in weight, a 3.0 tube for infants weighing 1-2 kg, and 3.5 or 4.0 tube for infants over 3 kg [10], however, current clinical guidelines do not make specific mention to the role of ETT leakage [7,11].

Uncuffed ETT are allowing an air leak during a peak inspiratory pressure, which provides a sufficient seal [8]. Although the use of uncuffed ETTs is still the standard practice in neonatal intensive care units, recently, microcuff ETTs have been introduced. These tubes minimize the ETT leak, providing efficient ventilation and decreasing the risk of pulmonary aspiration [9]. However, there are not enough studies available to compare the advantages of cuffed ETTs versus uncuffed ETTs in neonates [12-13]. The smallest microcuff ETT has an internal diameter of 3.0 mm, and the manufacturer recommends its use in term infants ≥ 3 kg [9].

The objective of this study was to compare the outcome of resuscitation by applying an ETT with less leakage, an ETT with moderate leakage, and an ETT with high leakage during mechanical ventilation in a swine model of prolonged perinatal asphyxia. The primary outcome variable was four hours post-resuscitation survival and secondary outcomes included: two hours post-resuscitation survival, time to ROSC, hemodynamic parameters, arterial oxygen saturation, and lactate levels.

## Materials and methods

We used a swine model of perinatal asphyxia, as newborn piglets are equivalent to human infants at 36-38 weeks of gestational age and have a comparable size and weight (1.5-2 kg body weight), allowing easy instrumentation to invasively monitor hemodynamic parameters, blood gases, and physiological measurements, as well as the ability to monitor the degree of hypoxia-asphyxia and reoxygenation in the recovery phase [14].

Α prospective, randomized, control laboratory study was performed between 2014 and 2015. Thirty Landrace/large white pigs, with conventional microbiological status, aged one to four days and weighting 1.754±218 gr, were used in this laboratory study. The animals were taken to the laboratory (Experimental, Educational and Research Center ELPEN) on the morning of the day of the experiment. All the animals came from the same farm, located in Koropi, Greece (Validakis farm). Pre-warmed padded boxes were used for their transport to the laboratory by a temperature-controlled truck within half an hour, so feeding was not required at the research facility.

Three strategies of ventilation were performed on piglets based on the selection of ETT size to achieve less, moderate, and high ETT leakage during their mechanical ventilation. ETT leak increases as the ETT diameter decreases and the significance of this relationship increases with an extended duration of mechanical ventilation [15]. The intubation that was performed with ETT no. 4 was defined as less-leakage ventilation, with ETT no. 3.5 was defined as moderate leakage ventilation and with no. 3 as high leakage ventilation.

The piglets were randomly allocated into three groups. Sealed envelopes with predetermined random allocation were used for the random allocation process. Each sealed envelope was assigning the piglets into three groups: a) group C (n=10) was intubated with a size 4.0 endotracheal tube (Portex, ID Smiths Medical, Keene, NH); b) group A (n=10) was intubated with a size 3.0 endotracheal tube (Portex. ID Smiths Medical, Keene. NH); and c) group B (n=10) was intubated with a size 3.5 endotracheal tube (Portex. ID Smiths Medical, Keene. NH). The experiment laboratory staff were entirely blinded to the allocation except for the veterinary scientist (vet) who intubated the piglets. All the piglets received standard cardiopulmonary resuscitation (CPR) as per 2015 European Resuscitation Council guidelines for neonatal resuscitation by the same operator who was carrying a Newborn Life Support certification (NI: NLS director) [16-17]. A data collection form based on predefined documentation was used for the collection of the data. The data analysis was performed by personnel who were blinded to group allocation.

An a priori power analysis was conducted for the primary outcome “four hours survival.” Due to the lack of previous studies, we assumed a large relative hazard (RH) of ETT with minimal leakage, compared to ETT with moderate to large leakage since a large RH would be clinically more meaningful (RH=6.7; a=0.05; power=0.80), which yielded the desired sample size of N=30 [18-19].

The experimental protocol was approved by the Greek General Directorate of Veterinary Services (permit number 1184 / 28.2.2014) according to Greek Legislation regarding scientific and experimental procedures (Presidential Decree 160/1991. in compliance with the European Directive 86/609 / EEC and in conformance with the European Convention for the protection of vertebrate animals used for experimental or other scientific purposes, 123/1986). The anesthetic and invasive procedures performed in the animals were guided by the Guide for the Care and Use of Laboratory Animals according to the principle of 3Rs [20]. The experimental protocol was also approved by the Ethics Committee of the Medical School of National and Kapodistrian University of Athens.

Protocol-preparatory phase

Originally, all animals were sedated with a single intramuscular injection of 10 mg/kg ketamine hydrochloride (Merial, Lyon, France). 0.5 mg/kg midazolam (Dormicum 1, Hoffmann-La Roche, Athens, Greece) and 0.01 mg/kg atropine sulfate (Efar, Athens, Greece). Anesthesia was induced with an intravenous bolus dose of 1 mg/kg propofol (Diprivan, AstraZeneca) and 10μg/kg fentanyl (Fentanyl, Janssen-Cilag) via the marginal auricular vein, which was catheterized by a 24G catheter (Jelco R. Smiths Medical, N. Papapostolou SA, Athens, Greece). The anesthetized piglets were intubated with an endotracheal tube (3, 3.5, or 4) (Portex, ID Smiths Medical, Keene, NH) via direct laryngoscopy as appropriate. Auscultation and capnography confirmed the proper location of the endotracheal tube. Infusion of 10 ml/kg/h of normal saline 0.9% and 5 ml/kg/h of dextrose in water 5% were initiated to prevent dehydration and hypoglycemia. Anesthesia, muscle relaxation, and analgesia were maintained with the infusion of 1 mg/kg of propofol, 0.15 mg/kg of cis-atracurium, and 1 mcg/kg of fentanyl. To ascertain synchrony with the ventilator, animals were administered intravenous fentanyl (10 mcg/kg) and cis-atracurium (0.15 mg/kg). The piglets were ventilated in pressure-controlled mode (Soxil, Soxitronic, Felino, Italy) with a tidal volume (VT) of 10-15 ml/kg at a pressure of 19cmH_2_O with a fraction of inspired oxygen (FiO_2_) of 0.21. The respiratory rate was adjusted to 30-40 breaths per minute in order to maintain end-tidal CO_2_ (ETCO_2_) of 35-40 mmHg. End-tidal CO_2 _(ETCO_2_) was monitored with a side-stream infrared CO_2_ analyzer (Datex Engstrom. Type TC 200-22-01 Instrumentarium, Helsinki, Finland) and pulse oximetry-monitored oxygen saturation (SpO_2_) throughout the experiment. Body temperature was maintained at 38+1^o^C with a table heating pad and an overhead heating lamp and was monitored with a thermometer (per rectum) by Matron, BPM 1000, VET. ET Medical Devices SpA (Cavareno TN, Italy).

The right internal jugular vein and the right carotid artery were catheterized, with single-lumen fluid-filled silicone catheters under aseptic conditions (S1UVC5.0. NeoCare®; Klein-Baker Medical Co. San Antonio, TX). For continuous monitoring of central venous pressure (CVP), systolic pressure (SP), mean arterial pressure (MAP), and diastolic pressure (DP) of the carotid artery, a normal saline-filled arterial catheter (model 6523, USCI CR, Bart. Papapostolou. Athens, Greece) was inserted into the aorta via the right carotid artery. A central vein catheter (Opticath 3.5 F. Abbott. Athens. Greece) was inserted into the right atrium via the right jugular vein for continuous measurement of systolic and diastolic right atrial pressures (Matron, BPM 1000, VET, ET Medical Devices SpA). Heart rate (HR) and ECG were also monitored continuously.

Experimental protocol

**Figure 1:**
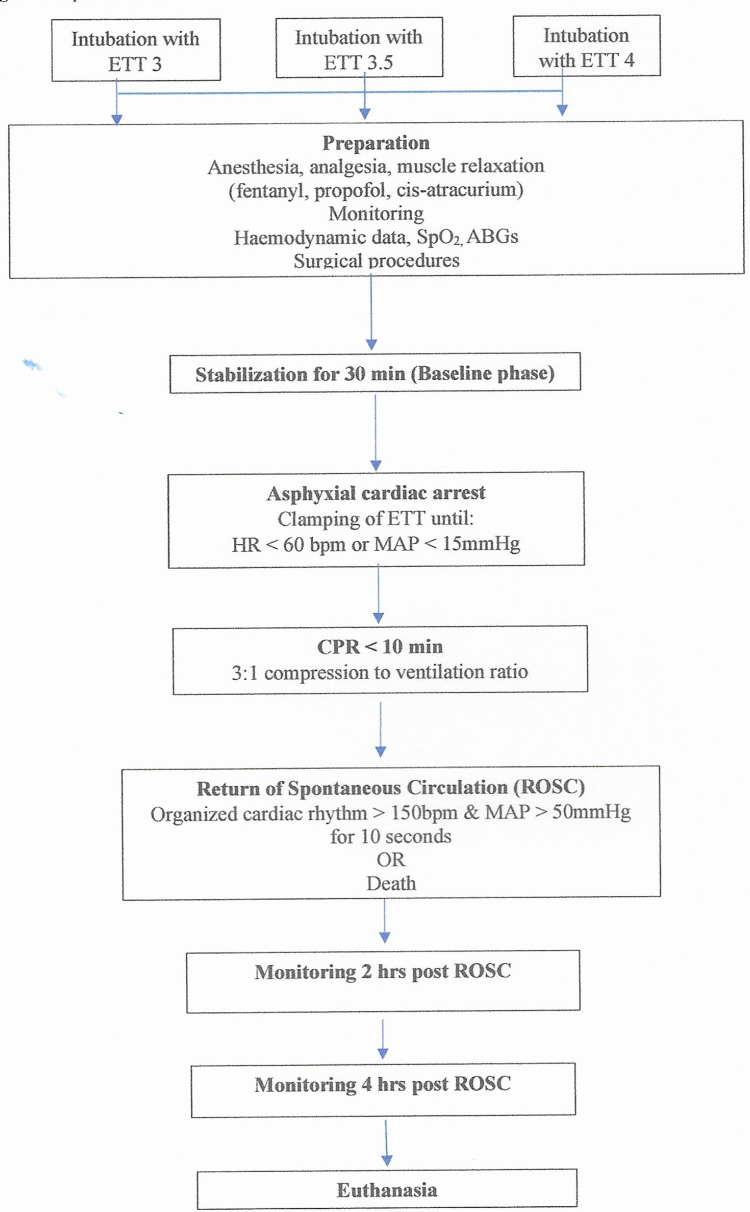
Experimental flowchart

The piglets were stabilized for 30 min and baseline hemodynamic data were collected (HP, SP, MAP, DP, arterial blood gases (ABGs)). Asphyxial cardiac arrest was induced by clamping of the endotracheal tube while the infusion of anesthetic drugs was stopped. The piglets were left untreated until signs of hemodynamic instability, defined as either bradycardia (HR<60 beats per minute (bpm) or severe hypotension (MAP<15mmHg) were detected. The time needed for each animal to develop signs of hemodynamic instability was recorded using a digital stopwatch. During hemodynamic instability, blood samples were taken to confirm hypoxemia (pO2=30-50 mmHg). Resuscitation was initiated according to the Newborn Life Support (NLS) algorithm and ventilation was performed with a NeopuffTM Infant Resuscitator (Fisher & Paykel Healthcare, Auckland, New Zealand) (IP: 30 cmH2O, positive end-expiratory pressure (PEEP): 5 cmH2O, gas flow meter: 8L/min) [18-19]. The effectiveness of the applied resuscitation efforts was assessed every 30 sec. No resuscitation drugs were administered during CPR. Endpoints of the experiment were defined as asystole or return of spontaneous circulation (ROSC). The piglets that achieved spontaneous circulation were monitored for four hours, under continuous anesthesia and mechanical ventilation. ROSC was defined as an increase in heart rate >150/min for 15 sec and the presence of a regular cardiac rhythm with a mean arterial pressure ≥60 mmHg for a minimum duration of 15 mins. For the study purposes, ROSC clinical status was characterized as the “survival” condition. All the animals that regained ROSC and survived were euthanized four hours later by intravenous administration of thiopental (140mg/kg), leading to rapid death. The experimental protocol is described in Figure 1.

Data collection and statistical analysis

Data analysis was based on predefined data points (baseline, hypoxia, ROSC, two hours post-ROSC, and four hours post-ROSC) on a prospective data collection form. The authors, as well as the whole experimental team (except vet), were blinded to measurements until the end of the study and the end of the statistical analysis.

Hemodynamic parameters, SpO_2_, arterial blood (ABGs) (pHOx plus C; Nova Biomedical. Waltham, MA, USA), and lactate levels were measured and recorded in all five time points of the experiment. The normality of the variables was initially tested using the Shapiro-Wilk criterion. The quantitative variables following normal distribution were expressed as mean±standard deviation and those that were not following normal distribution were described by median and percentiles (interquartile range: IQR). Qualitative variables are expressed as absolute and relative frequencies. Analysis of variance (ANOVA) was applied to detect differences between groups for variables that were following normal distribution and the Kruskal Wallis test to detect differences between groups for the variables that were not following a normal distribution. Wilcoxon signed-rank for paired data was used for variables that were not following normal distribution to assess the correlation between different time points in the same group. Repeated measurements ANOVA was used to evaluate potential differences between baseline, hypoxia, ROSC, two hrs post-ROSC, and four hours post-ROSC points to following normal distribution or Friedman’s test otherwise. Fisher's Exact test was used to compare categorical variables. Statistical significance was set at 0.05.

The statistical analysis was conducted using the SPSS version 26 software (IBM Corp., Armonk, NY) [21]. A Kaplan-Meier curve with the log-rank non-parametric test was applied to detect differences in survival between the study groups.

## Results

The baseline characteristics of the three groups are described in Table 1. Prior to the stabilization phase, one piglet from group A died. At baseline, there were no statistically significant differences in hemodynamic parameters among the three groups except for right atrial pressure (p=0.001) (group C had significantly increased right atrial pressure (5 mmHg) vs group A (2 mmHg) and group B (3 mmHg)). Regarding lactate levels, group A had significantly increased levels (1.7+0.23 mmol/l) vs group B (1.35+0.26 mmol/l) and group C (1.4+0.24 mmol/l) (p=0.001) (Table 1).

**Table 1:** Variables measurements at Baseline time in the three groups (A, B, and C) of piglets presented as means ± SD or median (IQR) *The mean difference is significant at the 0.05 level. SD: standard deviation; IQR: interquartile range; BP: blood pressure; bpm: beats per minute

Measurements Baseline	Group A (no 3) mean±SD/median (IQR)	Group B (no 3.5) mean±SD/median (IQR)	Group C (no 4) mean±SD/median (IQR)	P-value
Weight (g)	1722±314	1850±160	1690±180	0.25
Heart rate (bpm)	169.89±38.75	185.7±26.3	184±9.1	0.39
Systolic BP (mmHg)	78.67±9.2	83.5±15.37	81±3.9	0.61
Diastolic BP (mmHg)	47(19)	49(13.0)	53(14)	0.67
Mean BP (mmHg)	57.67±6.8	66.6±11.42	65.2±4.1	0.53
Right atrium (mmHg)	2(2.0)	3(1.0)	5(2.0)	0.001*
SpO2 (%)	97(2.5)	99(1.25)	98(2.25)	0.24
Arterial pH	7.42±0.12	7.4±0.05	7.38±0.05	0.53
Arterial pO2 (mmHg)	123.3±44.5	176.4±62.3	127.4±31.0	0.69
Arterial pCO2 (mmHg)	40±10.7	37.34±5.1	39.74±4.4	0.66
HCO3 (mmol/L)	25.15±2.4	23.9±4	23.05±2.99	0.37
Lactate (mmol/L)	1.7±0.23	1.4±0.24	1.35±0.26	0.01*
Duration of the asphyxia interval sec	383.3±211.24	547.3±224.43	445.3±226.03	0.279

There were no statistically significant differences in hemodynamic parameters during the hypoxia phase. The duration of the asphyxia interval (clamping of the tube until the event of bradycardia (HR<60 mmHg) or hypotension (MAP<15 mmHg)} ranged from 45-870 sec. Although there was a difference in the duration of asphyxia interval among the three groups, it was not statistically significant (p=0.279). Group B exhibited the longest time (547.3± 224.43 sec) to asystole vs group A (383.3.3 ± 211.24 sec) and vs group C (445.3±226.03) (Table 1).

Return of spontaneous circulation

**Figure 2:**
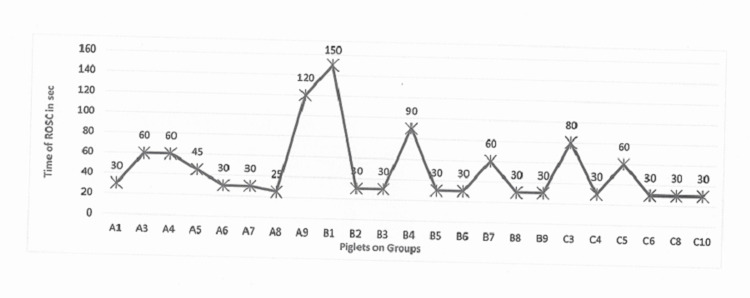
Time of return of spontaneous circulation in seconds per piglet in group A no. 3, group B no. 3.5, and group C no. 4

In total, 23 out of 29 piglets (80%) were successfully resuscitated. Four piglets from group C, one piglet from group A, and one from group B were not successfully resuscitated (Figure 2). The time to ROSC was not statistically significantly different among groups, with group C showing a trend for faster ROSC time (mean=43.33±21.6 sec), compared to group A (mean=50±31.5 sec) and group B (53.33±41.83 sec).

A statistically significant difference was noted in the right atrial pressure (RAP) between groups during ROSC (p=0.0001), with group C exhibiting higher right atrial pressure (4.5 mmHg) compared to group A (2 mmHg) and group B (2 mmHg) (Table 2). There was a difference in PO_2_ (p=0.032) between group B (mean=126.4±53.4 mmHg) compared to group A (mean=72.28±29.18 mmHg) and group C (mean=94.28±20.46 mmHg); SpO_2_ was statistically significantly different (p=0.003) in group B (mean=94.1±4.93) compared to group A (mean=76.22±17.34) and group C (mean=90.5±1.87) (Table 2).

**Table 2:** Variables measurements at return of spontaneous circulation (ROSC) time in the three groups (A, B, and C) of piglets presented as means ± SD or median (IQR) *The mean difference is significant at the 0.05 level. SD: standard deviation; IQR: interquartile range; BP: blood pressure; bpm: beats per minute

Variables at ROSC	Group A (no 3) mean±SD/median (IQR)	Group B (no 3.5) mean±SD/median (IQR)	Group C (no 4) mean±SD/median (IQR)	P-value
Heart rate (bpm)	201.63±40.29	208.78±49.89	223±26.23	0.63
Systolic BP (mmHg)	82.74±23.76	84.89±19.82	94.83±18.84	0.51
Diastolic BP (mmHg)	53.13± 18.8	56.22±23.9	47.83± 10.54	0.72
Mean BP (mmHg)	65.13±19.6	64.78±17.73	68.33±12.4	0.92
Right atrium (mmHg)	2(2)	2(1)	4.5(2)	0.001*
SpO2 (%)	76.22±17.34	94.1±4.93	90.5±1.87	0.003*
Arterial pH	7.02±0.16	7.06±0.11	7.1±0.15	0.56
Arterial pO2 (mmHg)	72.28±29.18	126.4±53.4	94.28±20.46	0.032*
HCO3 (mmol/L)	18.3±2.9	16.5±3.8	19.01±4.9	0.44
Arterial pCO2 (mmHg)	74.52±24.4	57.74±16.57	61.2±13.3	0.19
ROSC in sec	50±31.5	53.33±41.83	43.33±21.6	0.85
Lactate (mmol/L)	1.8(0.18)	2.05(0.35)	2.42(1.54)	0.062

Post-resuscitation measurements (two hours and four hours post-ROSC)

Heart rate (HR), diastolic pressure (DP), mean arterial pressure (MAP), and right atrium (RA) two hours post-ROSC, were found to be statistically significantly different (p<0.001) in group C compared to group A and group B (Table 3).

**Table 3:** Variables measurements at two hours post-ROSC phase in the three groups (A, B, and C) of piglets presented as means ± SD or median (IQR) *The mean difference is significant at the 0.05 level. SD: standard deviation; IQR: interquartile range; BP: blood pressure; bpm: beats per minute

Variables at 2h	Group A (no 3) mean±SD/median (IQR)	Group B (no 3.5) mean±SD/median (IQR)	Group C (no 4) mean±SD/median (IQR)	P-value
Heart rate (bpm)	160±14.76	177.62±20.77	232.5±8.48	0.022*
Systolic BP (mmHg)	52.37±19.3	66.87±12.6	79.5±19.1	0.09
Diastolic BP (mmHg)	24.87±8.9	35.87±12.75	45±0.01	0.046*
Mean BP (mmHg)	35.7±12.91	50±12.64	61.5±4.9	0.027*
Right atrium (mmHg)	2.5(2.5)	2(0.75)	2(0.7)	0.06
SpO_2_ (%)	92.83±8.09	97.35±1.89	97.5±2.12	0.27
Arterial pH	7.22(0.18)	7.35(0.17)	7.2(-)	0.64
Arterial pO_2_ (mmHg)	102.77±47.31	147.87±53.01	117.5±21.92	0.212
HCO_3_ (mmol/L)	20.25±3.8	21.6±5.3	21.4±2.96	0.83
Arterial pCO_2_ (mmHg)	51.05(11.35)	41.9(32.4)	40.05(-)	0.11
Lactate (mmol/L)	2.02(0.79)	2.05(0.31)	1.9(-)	0.861

No statistically significant difference was observed in hemodynamic parameters or ABGs between the three groups at four hours post-resuscitation. Lactate levels appeared statistically significant lower (p=0.034) in group C (mean=0.92±0.07 mmol/L) compared to group A (mean=1.13±0.1 mmol/L) and group B (mean=1.08±0.07 mmol/L) at four hours post-ROSC (Table 4).

**Table 4:** Variables measurements at four hours post the ROSC phase in the three groups (A, B, and C) of piglets presented as means ± SD or median (IQR) *The mean difference is significant at the 0.05 level. SD: standard deviation; IQR: interquartile range; BP: blood pressure; bpm: beats per minute

Variables at 4h	Group A (no 3) mean±SD/median (IQR)	Group B (no 3.5) mean±SD/median (IQR)	Group C (no 4) mean±SD/median (IQR)	P-value
Heart rate (bpm)	161.6±24.94	175.85±26.80	198.5±2.12	0.199
Systolic BP (mmHg)	54.71±19.47	64.28±18.93	76±5.65	0.343
Diastolic BP (mmHg)	26±8.5	30.57±12.84	45.5±3.5	0.106
Mean BP (mmHg)	36.57±11.81	42.57±15.28	58.5±0.7	0.152
Right atrium (mmHg)	2(2)	2(0.75)	3.5(-)	0.091
SpO_2_ (%)	92.94±5.5	97.129±1.84	97±0	0.15
Arterial pH	7.23±0.074	7.32±0.11	7.35±0.021	0.132
Arterial pO_2_ (mmHg)	97.5±57.7	131.95±45.3	132.85±38.53	0.42
HCO_3_ (mmol/L)	24.32±5.9	22.85±2.64	21.75±2.3	0.7.17
Arterial pCO_2_ (mmHg)	56.87±10.68	44.65±12.29	52±1.41	0.157
Lactate (mmol/L)	1.13±0.1	1.08±0.07	0.925±0.077	0.034*

Survival

**Figure 3:**
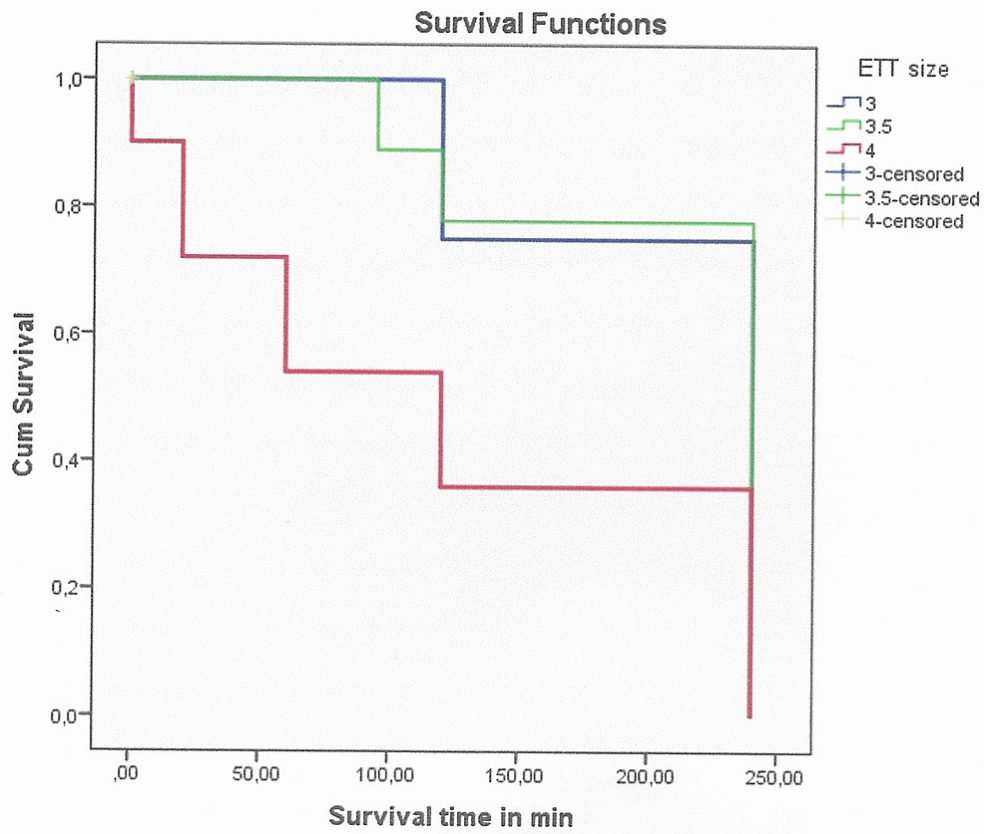
Kaplan-Meier survival graphs for groups A and C as well for groups B and C after CPR in minutes (p<0.001) CPR: cardiopulmonary resuscitation

All resuscitated animals (achieved ROSC) survived at two hours post-ROSC. At four hours post-ROSC, two (20%) animals achieved survival from group C, six (66.6%) piglets from group A, and six (60%) piglets from group B. Most of the surviving animals were intubated with an endotracheal tube size of 3.5 (group B) and 3 (group A). The log-rank control of Kaplan-Meier showed a statistically longer overall survival time in group A animals (mean=186,66±29.059 min) and group B animals (mean=189.5±27.391 min) than group C animals (mean=68±31.156 min) (x^2^=8.98, respectively, p=0.011) (Figure 3).

## Discussion

This is the first study, to our knowledge, which investigates the effect of different ETT sizes that prevent or allow endotracheal tube leakage, in resuscitation outcomes, and in the recovery of asphyxiated piglets. The most important findings were an increased likelihood of four hours survival in piglets with moderate and high ETT leakage, a statistically significant difference between groups during ROSC in PO_2_, in right atrial pressure, and SpO_2 _and in arterial pressure and other hemodynamic parameters at two hours post-ROSC. Also, lactate levels were statistically significantly lower at four hours post-ROSC. Those animals who were intubated with smaller ETTs achieved higher survival rates, which provides preliminary evidence of an association between leak tube ventilation and a successful resuscitation outcome [15].

In our study, in the group with minimal leakage compared to the groups with leakage, fewer piglets were successfully resuscitated and survived past two hours post-ROSC, suggesting that effective respiratory support and survival can be achieved with ETT leakage ventilation. Therefore, more investigation is needed on the effect of a tightly sealed endotracheal tube on resuscitation and longer-term outcomes in perinatal asphyxia.

Although tightly sealed ETTs may achieve higher tidal volumes and benefits in oxygenation, they may also increase peak inspiratory and mean airway pressures, as well as right atrial pressures, leading to barotrauma, lung interstitial edema, and compromised cardiac output [22]. This is supported by the increased RA pressures observed in our “no leak” group; however, we did not assess airway pressures, which may be linked to longer-term outcomes in neonates. Positive pressure ventilation (PPV) can increase the RAP, resulting in reduced venous return, right ventricular preload, lower cardiac output, and the survival of the ventilated subjects adversely [23-25]. Furthermore, Hentschel, et al. determined in vitro the pressure-flow characteristics of pediatric endotracheal tubes by measuring the pressure drop (ΔPΕΤΤ). They found that ΔPETT is relevant to the ETT size, with the narrower tubes leading to greater resistance in gas flow and vice versa. Therefore, it is presumable that in the “moderate” and “high leak” groups due to the application of smaller ETT sizes, more resistance was created in the gas flow, which allowed less pressure in the lungs and the right atrial, improving the survival rates [26].

Furthermore, the relatively shorter time of achieving ROSC that we observed in the “moderate leak” group may be associated with the hemodynamic benefits of moderate ETT leak and more efficient myocardial perfusion. Here it is important to note that the duration of hypoxia is directly proportional to the degree of brain damage, therefore moderate ETT leakage may also potentially minimize brain damage [27]. Yang et al. designed a clinically relevant model of perinatal asphyxia in rats to investigate the acute changes in brain pathology and the prolonged neurological impairments following this global ischemic injury. According to their experimental model, perinatal hypoxia leads to neuronal damage and apoptosis in the hippocampus [28].

The observed trend for higher PO_2_ and SpO_2_ in the “moderate leak” group suggests that better oxygenation can be achieved through mechanical ventilation with moderate ETT leakage compared to high or minimal leakage ventilation. Perhaps the leak around the ETT and chest compressions play a relevant role. Mendler et al. suggested that alveolar air may be exhaled via the leak caused by the cardiac compressions immediately after the lung inflation, resulting in falsely low measured tidal volumes. They noted that in hypoxic piglets, the ETT leak is increasing during chest compressions in ventilation modes with high PEEP [29]. Similar findings are reported by Li, et al. [30]. However, in our study, we did not measure the degree of leakage during the ventilation in any stage, neither during the chest compressions. The delivery of PEEP was low in all groups and remained unchanged during all stages of the study.

Thomas et al. suggested the selection of the right size of ETT by using the Sherman ratio. The right ETT will ensure an appropriate ‘leak on intubation, and it will achieve adequate ventilation while avoiding the side effects of unduly large ETTs, minimizing trauma during intubation, avoiding recurrent extubations/intubations [8].

## Conclusions

Perinatal asphyxia is characterized by intermittent periods of hypoxia-ischemia, which, if prolonged and intense enough, may cause irreversible damage. Our study indicates that intubation with an ETT leak significantly improved ROSC and survival in a porcine model of neonatal resuscitation. In the group with minimal leakage, compared to the groups with leakage, fewer piglets were successfully resuscitated and survived past two hours post-ROSC, suggesting that effective respiratory support and survival can be achieved with ETT leakage ventilation.

These results may suggest that the use of ETT with leakage during resuscitation can lead to better outcomes. The future replication of similar models will contribute to the accumulation of an important body of evidence that can inform oxygenation practices in humans during perinatal asphyxia and improve the outcomes of care.
